# Isolated Effects of Bone, Spring and Surgical Parameters on the Surgical Outcome in Spring‐Assisted Sagittal Synostosis Correction

**DOI:** 10.1049/htl2.70028

**Published:** 2025-11-12

**Authors:** Jenson Jacob, Selim Bozkurt

**Affiliations:** ^1^ School of Engineering Ulster University Belfast UK

**Keywords:** surgical outcomes, sagittal synostosis, spring‐assisted cranioplasty

## Abstract

Sagittal synostosis can be corrected using spring‐assisted cranioplasty which, involves removing bony parts from the skull and inserting compressed springs to expand the skull. However, surgical outcomes in spring‐assisted cranioplasty may remain suboptimal due to the interacting effects of skull, and spring parameters and surgical settings. Therefore, understanding the effects of skull and spring parameters, and surgical settings, on surgical outcomes is crucial to improving spring‐assisted cranioplasty. The aim of this study is to evaluate the isolated effects of calvarial bone and spring parameters and surgical settings on the surgical outcome in the correction of sagittal synostosis with spring‐assisted cranioplasty. A 3D parametric skull model with sagittal synostosis was used to simulate spring‐assisted cranioplasty. Computational simulations were used to simulate the effects of spring, surgical, and skull parameters. Results showed that relatively low elastic modulus and thickness in the calvarial bones had remarkable effects on surgical outcomes in sagittal synostosis after spring‐assisted cranioplasty. However, after spring‐assisted cranioplasty, the post‐operative cranial index increased a little for higher elastic modulus and thickness in the calvarial bones. Therefore, evaluating the mechanical and geometric properties of the skull during surgical planning may ensure successful surgical outcomes in sagittal synostosis treated with spring‐assisted cranioplasty.

## 1 Introduction

Craniosynostosis is a craniofacial disorder in which fibrous tissues between the bones of an infant's skull close prematurely before the skull and brain are fully developed [1]. The most common type of craniosynostosis is sagittal synostosis, and it comprises around 50% of craniosynostosis conditions in patients [2].

Sagittal synostosis can be treated using endoscopic methods, which aim to correct skull deformities over time, relying on skull growth [2]. Endoscopic strip craniectomy is used in patients between four and six months and is followed by post‐operative helmet therapy to improve the surgical outcome [3]. Spring‐assisted cranioplasty is a relatively new technique used to correct sagittal synostosis in patients younger than six months old [4]. Spring‐assisted craniosynostosis correction was initially done at Sahlgrenska University Hospital as a post‐operative method [5]. In this technique, springs are compressed at both ends and inserted into the skull to expand it, relying on spring forces [6]. Spring‐assisted cranioplasty in sagittal synostosis is a minimally invasive procedure resulting in reduced blood loss and requiring relatively short operative times and hospital stays compared to other methods used to correct sagittal synostosis [7, 8]. Therefore, it has gained popularity as a correction method in sagittal craniosynostosis [8]. Spring‐assisted cranioplasty involves making small incisions on the scalp to access the fused sagittal suture. It is followed by a craniotomy to remove a segment of bone along the suture. Metallic springs, which apply distractive forces to increase the width of the skull, are placed on the parietal bones [9, 10]. Pre‐operative helmeting may be used between the pre‐operative scan and surgery to reduce deterioration in head shape because of skull growth [11]. Pre‐operative surgical planning in craniosynostosis is a critical step to reduce operative times and duration of stay after the surgical operation and aims to optimise surgical outcome [12]. Computer tomography images are used to evaluate the severity and extent of the cranial deformity whilst confirming the diagnosis [13]. Patient‐specific 3D skull models can also be manufactured with rapid prototyping and 3D printing technologies to evaluate patient anatomy and may be utilised to evaluate surgical procedures (14). Despite the use of advanced clinical imaging modalities and digital tools, the outcome of spring‐assisted correction of sagittal synostosis may remain suboptimal due to the interaction of the skull and spring [15]. Also, spring‐assisted sagittal synostosis correction may cause neurological incidents or require a follow‐up operation to improve surgical outcomes [16, 17]. Therefore, further research is needed to improve its efficiency as a skull correction method [3, 18].

Computational modelling and simulations have been utilised as research tools to understand the implications and predict the outcome of spring‐assisted cranioplasty in patients with sagittal synostosis [19]. Borghi et al. [20] developed a patient‐specific model of spring‐assisted correction to evaluate sagittal synostosis. Cross et al. [21] used numerical simulations to compare various correction techniques, including spring‐assisted correction in sagittal synostosis. A computational modelling framework was developed to simulate skull growth and improve the management of sagittal synostosis [22]. Computational modelling was also used to simulate spring‐assisted cranioplasty over a range of parameters and utilised machine learning to predict surgical outcomes. [23] The surgical outcome in spring‐assisted cranioplasty depends on different parameters, such as the mechanical and geometric properties of the skull and spring, and surgical parameters [19, 23, 24]. Therefore, numerical simulations have also been used to understand the sensitivity of the surgical outcome on the bone, spring, and surgical parameters. For instance, finite element modelling and statistical methods were utilised to evaluate interrelations between surgical outcome and bone, spring, and surgical parameters in sagittal synostosis patients treated with spring‐assisted cranioplasty [25]. Such studies provide information about the global sensitivity of the surgical outcome depending on various parameters. However, variations in the mechanical and geometric properties of the skull and surgical settings affect the surgical outcome [26]. Therefore, the isolated effects of the bone, spring, and surgical parameters on the surgical outcome in spring‐assisted correction of sagittal synostosis must be understood to achieve optimal results after spring implantation. The aim of this study is to evaluate the isolated effects of bone, spring, and surgical parameters on the surgical outcome in the correction of sagittal synostosis with spring‐assisted cranioplasty.

## 2 Materials and Methods

**FIGURE 1:**
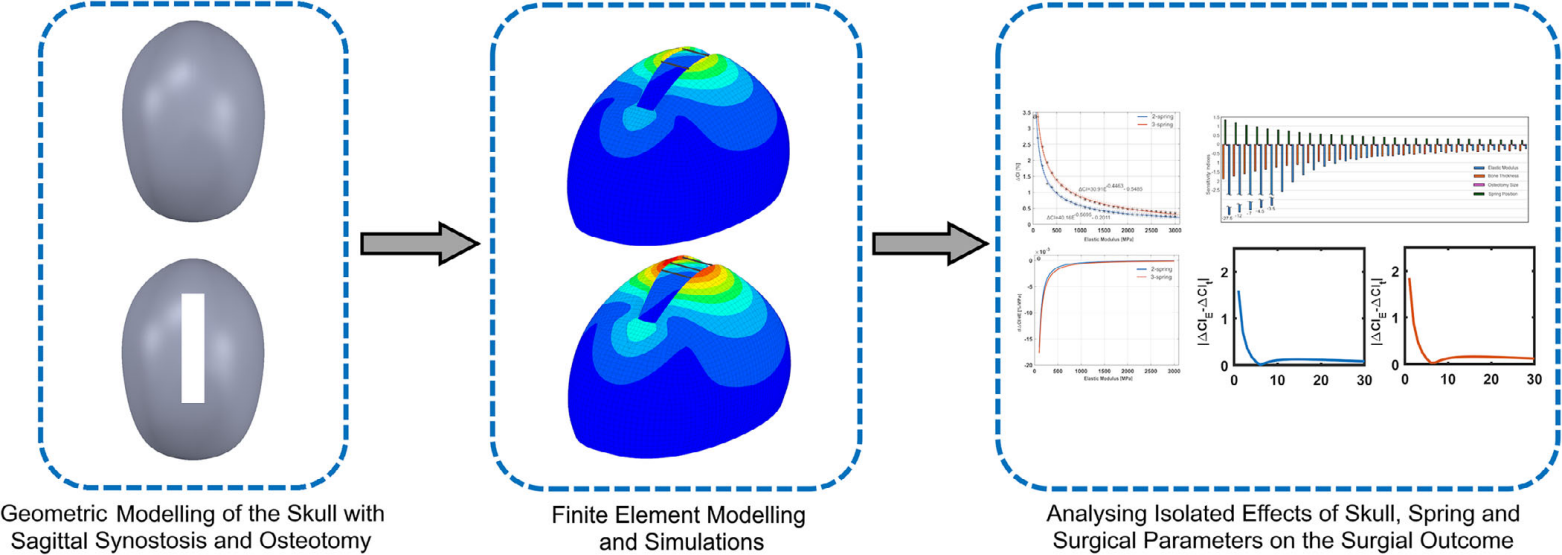
The overview of the framework for the modelling and simulation of spring‐assisted cranioplasty and evaluation of the effects of skull, spring, and surgical parameters on the cranial index.

In this study, a 3D parametric skull model with sagittal synostosis was used to simulate spring‐assisted cranioplasty. Finite element analyses were used to simulate the effects of the elastic modulus and thickness of the calvarial bones, spring position, osteotomy size, number of springs, and spring stiffness on the post‐operative cranial index after spring‐assisted cranioplasty. The influence of the above‐mentioned parameters on the change in the cranial index after spring‐assisted cranioplasty was evaluated by calculating local sensitivity indices. Direct comparison of the influence of the elastic modulus and thickness of the calvarial bones, spring position, and osteotomy size on the cranial indexes was evaluated using absolute differences between the change in the cranial indexes for the abovementioned parameters. The overview of the framework for the modelling and simulation of spring‐assisted cranioplasty and evaluation of the effects of skull, spring, and surgical parameters on the cranial index is given in Figure 1.

### 2.1 Geometric Modelling of the Skull With Sagittal Synostosis

The skull model used in this study was obtained from [23] with Creative Commons Attribution License (CC BY 4.0), which permits unrestricted use, distribution, and reproduction in any medium, provided the original author and source are credited. The 3D skull model represents a skull from a 5‐month‐old patent with sagittal synostosis. The length, breadth and height of the developed skull model were around 165 mm, 116 mm, and 87 mm [27] whereas the cranial index in the skull model was around 0.70. Coronal and lambdoid sutures on the skull model were created considering anatomical features, as given in [28] using MSC Marc 2022 (Hexagon, Stockholm, Sweden). Detailed information about the skull model can be found in [23].

### 2.2 Finite Element Modelling and Analysis of Spring‐Assisted Cranioplasty

Finite element modelling using MSC Marc 2022 Hexagon, Stockholm, Sweden) was done to simulate the isolated effects of elastic modulus and thickness of the calvarial bones, spring position, number of the springs on the skull and spring stiffness, and osteotomy size on the increase of cranial index after spring‐assisted cranioplasty. The effects of the bone, spring, and surgical parameters on the change in the cranial index after spring‐assisted cranioplasty were evaluated by changing the values of each parameter within the described range whilst keeping the other parameters constant using a similar described in [29]. The number of elements and nodes was determined after performing a mesh independent test, as given in [23]. Skull models were meshed using hexahedral quadratic elements. The numbers of elements and nodes were around 7500 and 42,000.

The reported range of the elastic modulus of the bones in a skull with sagittal synostosis is between 100 MPa and 4500 MPa in patients around five months old [24, 30, 31]. Therefore, the range of the calvarial bone elastic modulus in the simulations was between 100 MPa and 3000 MPa with 100 MPa intervals. The range of the parietal bone thickness is between 1.5 mm and 3 mm for six‐month‐old infants [32]. Also, the average parietal bone and frontal bone thicknesses change between 3.4 mm and 3.7 mm during the first six months of age in infants [33]. The range of bone thickness was between 2 mm and 4 mm, with 0.2 mm intervals in the skull model. 10 mm and 20 mm osteotomy sizes were reported for spring‐assisted cranioplasty in the literature [20, 34]. Therefore, the size of osteotomies changed between 10 mm and 20 mm, with 2 mm intervals in the simulations. Osteotomies were modelled by removing the bony parts between the coronal and lambdoid sutures [35]. Anterior and posterior springs are positioned 10 mm to 40 mm from the sutures [9, 34]. Therefore, the range for the spring positions from the sutures was defined between 10 mm and 40 mm with 3 mm intervals. Three different spring characteristics depending on the wire diameter were simulated as described by Borghi et al. [36]. The spring diameters were 1 mm, 1.2 mm and 1.4 mm, whereas the corresponding spring stiffnesses were 0.17 N/mm, 0.39 N/mm and 0.69 N/mm, respectively [36]. Spring‐assisted cranioplasty was simulated using two or three springs, as reported in the literature [37]. The elastic modulus and Poisson ratio of fibrous tissues, which included sutures and fontanelles, were 16 MPa and 0.49 in all the simulations [20, 38]. Fixed displacement boundary conditions were applied at the base of the skull model. The control values of the variables evaluated in the finite element simulations [20] are given in Table 1.

**TABLE 1:** The control values of the variables evaluated in the finite element simulations [20].

*E* [MPa]	*ν*	*t* [mm]	SP [mm]	OS [mm]	*k* [N/mm]
421	0.22	2	34	20	0.39

### 2.3 Relationships between the Skull, Spring, and Surgical Parameters and Change of the Cranial Index After Spring‐Assisted Cranioplasty

The relationships between the change in the cranial index after spring‐assisted cranioplasty and each parameter were developed using the curve fitting tool in MATLAB 2021b (MathWorks, Natick, Massachusetts, USA). A power function, as given below, was used to describe the relationships between the change in the cranial index after spring‐assisted cranioplasty (*ΔCI*) and the elastic modulus of the bones, bone thickness and spring positions (*X*).

(1) $$\Delta CI=AX^{B}+C$$

The relationship between the change in the cranial index after spring‐assisted cranioplasty (ΔCI) and osteotomy size (X) was described using a linear polynomial function, as given below.

(2) $$\Delta CI=P_{1}X+P_{2}$$

Change of the cranial index change after spring‐assisted cranioplasty with respect to the elastic modulus of the bones, bone thickness, spring positions, and osteotomy size (*dΔCI/dX*) was used to evaluate the change rate of the cranial index change with respect to the calvarial bone elastic modulus and bone thickness, spring position and osteotomy size after spring‐assisted cranioplasty. The regression equations were defined by testing different models and the goodness of the fit curve considering the coefficients of determination (*R^2^*).

### 2.4 Influence of the Skull, Spring, and Surgical Parameters on the Change of the Cranial Index after Spring‐Assisted Cranioplasty

The fit curves describing the relationships between the change in the cranial index and skull, spring, and surgical parameters were used to generate data samples within the range of the parameters. Thirty data samples with equal intervals were generated for calvarial bone elastic modulus and thickness, spring position, osteotomy size, and corresponding cranial indexes for assisted cranioplasty with two and three springs. Sensitivity indices for each step were calculated using a similar method to indirect local sensitivities as described in [39], and given below.

(3) $$S_{i}=\frac{\Delta CI_{n+1}-\Delta CI_{n}}{\Delta X}$$

(4) $$\Delta X=\frac{\left(X_{n+1}-X_{n}\right)}{\left(X_{\max}-X_{1}\right)}$$

Here, *S_i_* represents sensitivity indices, *ΔCI* is the change in the cranial index after spring‐assisted cranioplasty, *ΔX* represents the normalised difference of the evaluated skull, spring, or surgical parameter. Subscripts *n* and *max* represent the step number and the maximum step.

The effect of elastic modulus, bone thickness, spring position, and osteotomy size on the change of cranial index after was also compared, calculating the absolute differences between the change of the cranial indexes for each parameter as given below.

(5) $$\Delta CI_{x-y}=\left|\Delta CI_{x,i}-\Delta CI_{y,i}\right|$$

Here, *ΔCI* is the change of the cranial index after spring‐assisted cranioplasty, and subscripts *x*, *y*, and *i* represent the evaluated skull, spring, or surgical parameters and a number of the generated data points from the fit models.

## 3 Results

**FIGURE 2:**
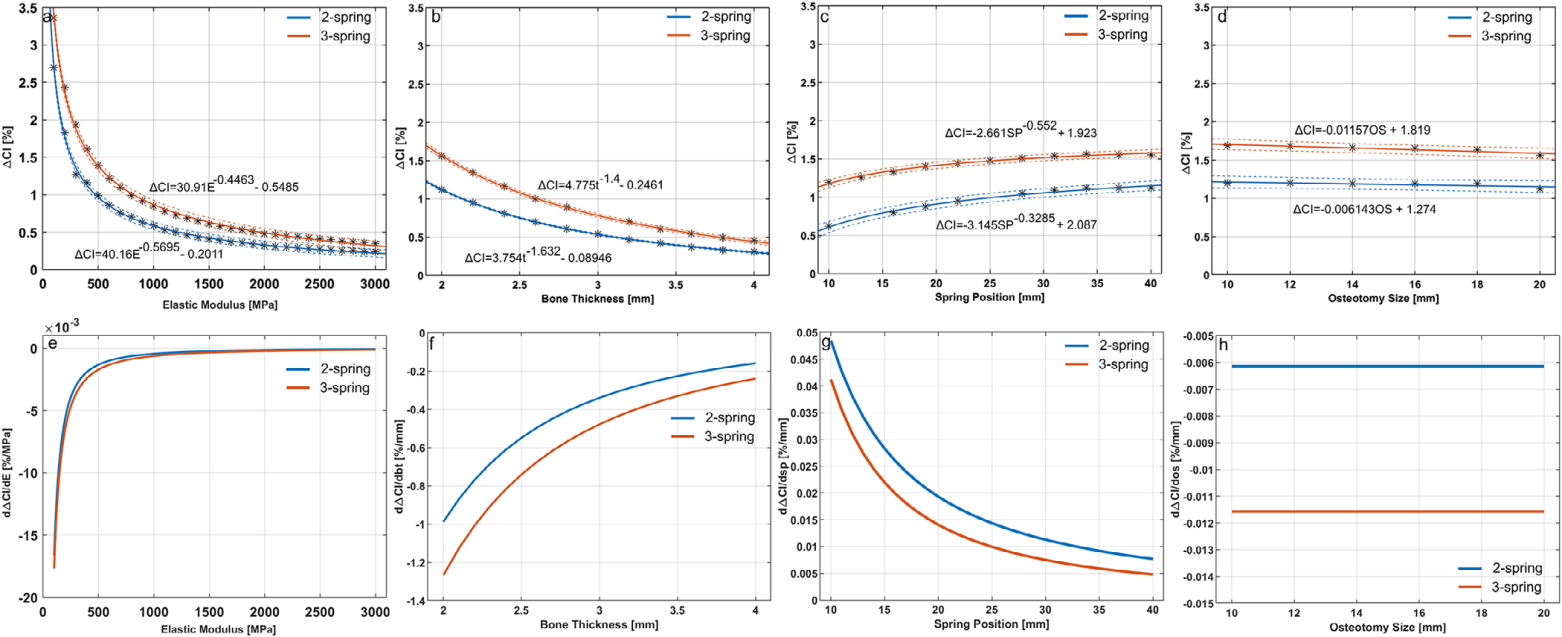
The relationships between the increase in the cranial index after spring‐assisted cranioplasty and the elastic modulus (a) and thickness of the bones (b), spring position (c) and osteotomy size (d) and the change rate of the increase in the cranial index after spring‐assisted cranioplasty with respect to elastic modulus (e) and thickness of the bones (f), spring position (g) and osteotomy size (h), (dashed lines show 95% confidence intervals).

The relationships between the increase in the cranial index after spring‐assisted cranioplasty and the elastic modulus and thickness of the bones, spring position, and osteotomy size and the change rate of the increase in the cranial index after spring‐assisted cranioplasty with respect to elastic modulus and thickness of the bones, spring position, and osteotomy size with 95% confidence intervals are given in Figure 2. The coefficients in the fit models and the *R* ^2^ are given in Table 2.

**TABLE 2:** The coefficients in the fit models describing the relationships between the increase in the cranial index after spring‐assisted cranioplasty (*ΔCI*) and the elastic modulus (*E*) and thickness of the bones (*t*), spring position (*SP*), and osteotomy size (*OS*) and coefficients of determination (*R^2^*). *A*, *B*, and *C* are the coefficients in the power functions, and *P_1_* and *P_2_* are the coefficients in the polynomial functions.

–	–	*A*	*B*	*C*	*P_1_*	*P_2_*	*R^2^*
ΔCI—E	2‐spring	40.16	−0.5695	−0.2011	—	—	0.998
	3‐spring	30.91	−0.4463	−0.5485	—	—	0.9985
ΔCI—t	2‐spring	3.754	−1.632	−0.08946	—	—	0.9998
	3‐spring	4.775	−1.4	−0.2461	—	—	0.9995
ΔCI—SP	2‐spring	−3.145	−0.3285	2.087	—	—	0.9887
	3‐spring	−2.661	−0.552	1.923	—	—	0.9862
ΔCI—OS	2‐spring	—	—	—	−0.006143	1.274	0.564
	3‐spring	—	—	—	−0.01157	1.819	0.856

Calvarial bones with a relatively low elastic modulus caused a relatively high increase in the cranial index after spring‐assisted cranioplasty. Similarly, thinner calvarial bones caused a relatively high increase in the cranial index after spring‐assisted cranioplasty. Positioning springs further from the coronal and lambdoid sutures resulted in a relatively high increase in the cranial index after spring‐assisted cranioplasty. The increase in the cranial index after spring‐assisted cranioplasty reduced with increasing osteotomy size. The use of three springs to correct sagittal synostosis resulted in a relatively high increase in the cranial index after spring‐assisted cranioplasty.

Change of the increase in the cranial index with respect to the calvarial bone elastic modulus (*dΔCI/dE*) becomes relatively stable for the calvarial bone elastic modulus values higher than 900 MPa. The negative change in the cranial index with respect to the calvarial bone elastic modulus (*dΔCI/dE*), thickness (*dΔCI/dt*), and osteotomy size (*dΔCI/dOS*) shows an inverse relation between the change in the cranial index after spring‐assisted cranioplasty and the parameters above. Change of the increase in the cranial index with respect to osteotomy size (*dΔCI/dOS*) was constant with increasing osteotomy size after spring‐assisted cranioplasty.

**FIGURE 3:**
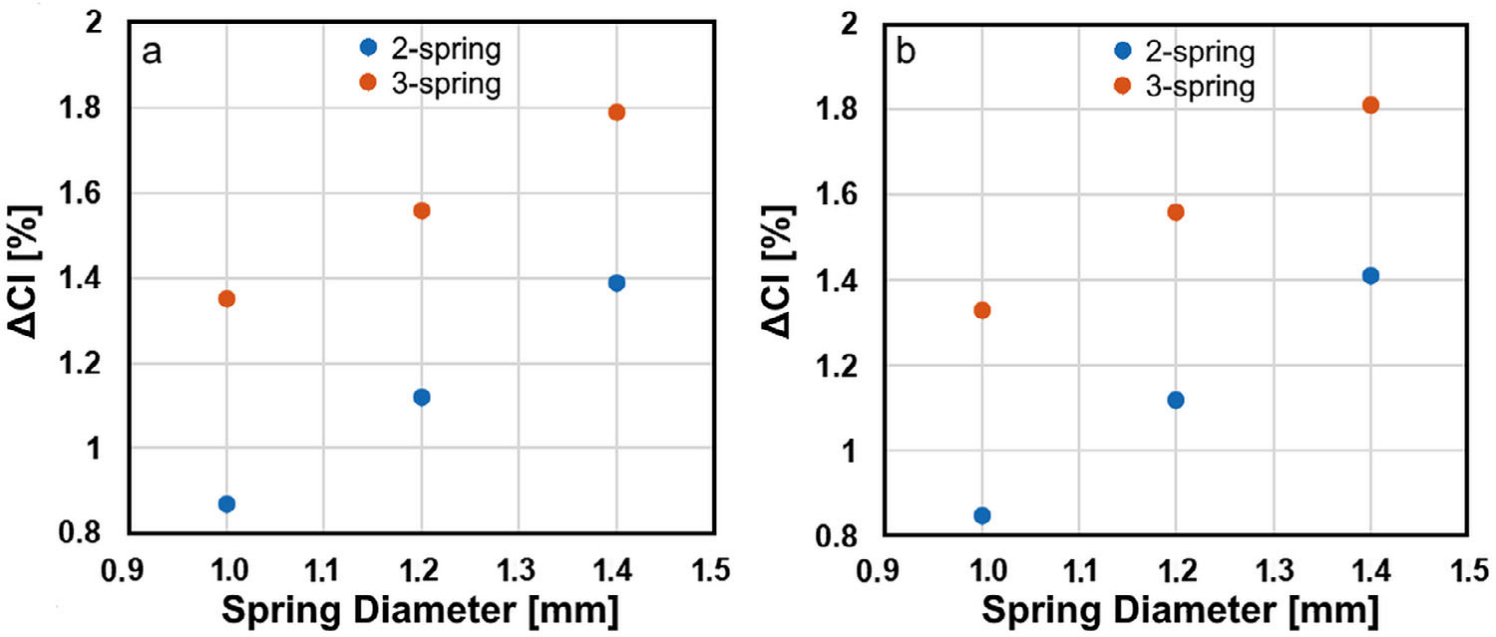
Change in the cranial index after positioning a 1.2 mm diameter spring near the lambdoid suture and implanting 1 mm, 1.2 mm and 1.4 mm springs near the coronal suture (a), change in the cranial index after positioning a 1.2 mm diameter spring near the coronal suture and implanting 1 mm, 1.2 mm, and 1.4 mm springs near the lambdoid suture (b). The third spring was implanted between the springs near the coronal and lambdoid sutures and was a spring with a 1.2 mm diameter.

The *R* ^2^ was higher than 0.98 in all the fit models except for the regression lines describing the relationships between the increase in the cranial index and osteotomy size. The *R^2^* in these models remained below 0.90 for spring‐assisted cranioplasty with 2 and 3 springs. The effect of the spring diameter on the change in the cranial index after spring‐assisted cranioplasty is shown in Figure 3.

Relatively high spring diameters caused a higher increase in the cranial index after spring‐assisted cranioplasty. Anterior and posterior springs with different characteristics caused a minimal difference in the increase in the cranial index after spring‐assisted cranioplasty. Again, implanting three springs resulted in relatively high changes in the cranial index after spring‐assisted cranioplasty.

**FIGURE 4:**
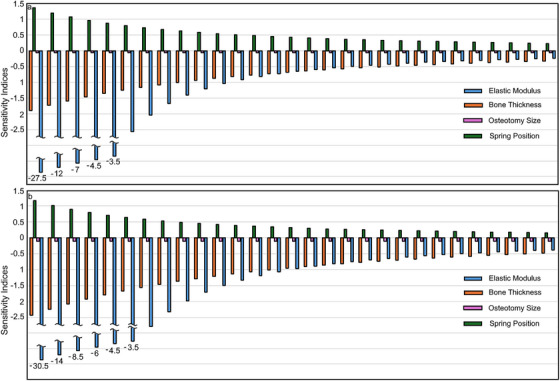
The sensitivity of the cranial index with around 3.45% step size in the calvarial bone elastic modulus and thickness, spring position, and osteotomy size: (a) spring‐assisted cranioplasty with 2 springs; and (b) spring‐assisted cranioplasty with 3 springs.

Sensitivity indices for the change in the cranial index with around 3.45% step size in the calvarial bone elastic modulus and thickness, spring position and osteotomy size are given in Figure 4.

Negative sensitivity indices show an inverse relation between the change in the cranial index after spring‐assisted cranioplasty and the evaluated parameters. The magnitudes of the sensitivity indices were remarkably high for relatively low calvarial bone elastic moduli. Increasing the elastic moduli reduced the magnitude of the sensitivity indices remarkably. Magnitudes of the sensitivity indices decrease with increasing bone thickness. Similarly, the magnitudes of the sensitivity indices decrease with positioning the anterior and posterior springs further from the coronal and lambdoid sutures. However, sensitivity indices showing the effect of the spring position are positive and indicate a direct relationship between the spring position and the increase in the cranial index after spring‐assisted cranioplasty. The magnitudes of the sensitivity indices showing the effect of the osteotomy size on the change of the cranial index after spring‐assisted cranioplasty remain constant. The magnitudes of the sensitivity indices are relatively high in the skull model corrected with three springs, except for the sensitivity indices for the spring position. The average sensitivity of the change in the cranial index with around 3.45% step size in the calvarial bone elastic modulus and thickness, spring position, and osteotomy size is given in Table 3.

**TABLE 3:** The average sensitivity of the change in the cranial index with around 3.45% step size in the calvarial bone elastic modulus and thickness, spring position, and osteotomy size.

—	ΔCI‐E	ΔCI‐t	ΔCI‐SP	ΔCI‐OS
2‐spring	−2.49	−0.82	0.54	−0.06
3‐spring	−3.09	−1.12	0.41	−0.12

**FIGURE 5:**
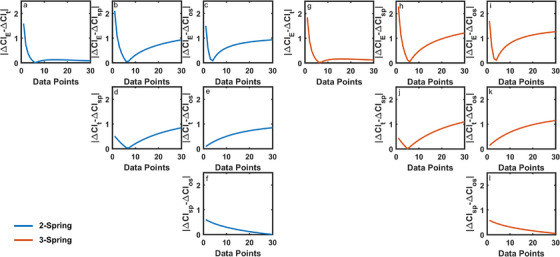
Absolute differences between the change in the cranial indexes after spring‐assisted cranioplasty for the calvarial bone elastic modulus and bone thickness, spring position, and osteotomy size. *ΔCI* represents the change in the cranial index after spring‐assisted cranioplasty; subscripts *E*, *t*, *SP*, and *OS* represent calvarial bone elastic modulus and bone thickness, spring position, and osteotomy size, respectively.

The change in the cranial index after spring‐assisted cranioplasty was more sensitive to changes in the elastic modulus of the calvarial bones in comparison to the changes in calvarial bone thickness, spring position, and osteotomy size (Table 3). The magnitude of the average sensitivity of the change in the cranial index was relatively high in the skull model corrected with three springs, except for the average sensitivity index for the spring position. Absolute differences between the change in the cranial indexes after spring‐assisted cranioplasty for the calvarial bone elastic modulus and bone thickness, spring position, and osteotomy size are given in Figure 5.

Initially, the absolute difference between the changes in the cranial index due to changes in the elastic modulus and the thickness of the calvarial bones decreases. After reducing to a minimal value, it slightly increases (Figure 5a, g). The absolute difference between the changes in the cranial index due to changes in the elastic modulus of the calvarial bones and spring positions decreases at relatively low elastic modulus and spring position values. After decreasing to a minimal value, it increases (Figure 5b, h). There is a similar change in the absolute difference between the increase of the cranial index due to changes in the elastic modulus of the calvarial bones and osteotomy size (Figure 5c, i).

The absolute difference between the changes in the cranial index due to changes in the thickness of the calvarial bones and spring positions decreases at relatively low bone thickness and spring position values. After reaching a minimal value, it increases (Figure 5d, j). The absolute difference between the changes in the cranial index due to changes in the thickness of the calvarial bones and osteotomy size increases with increasing bone thickness and osteotomy size (Figure 5e, k).

**FIGURE 6:**
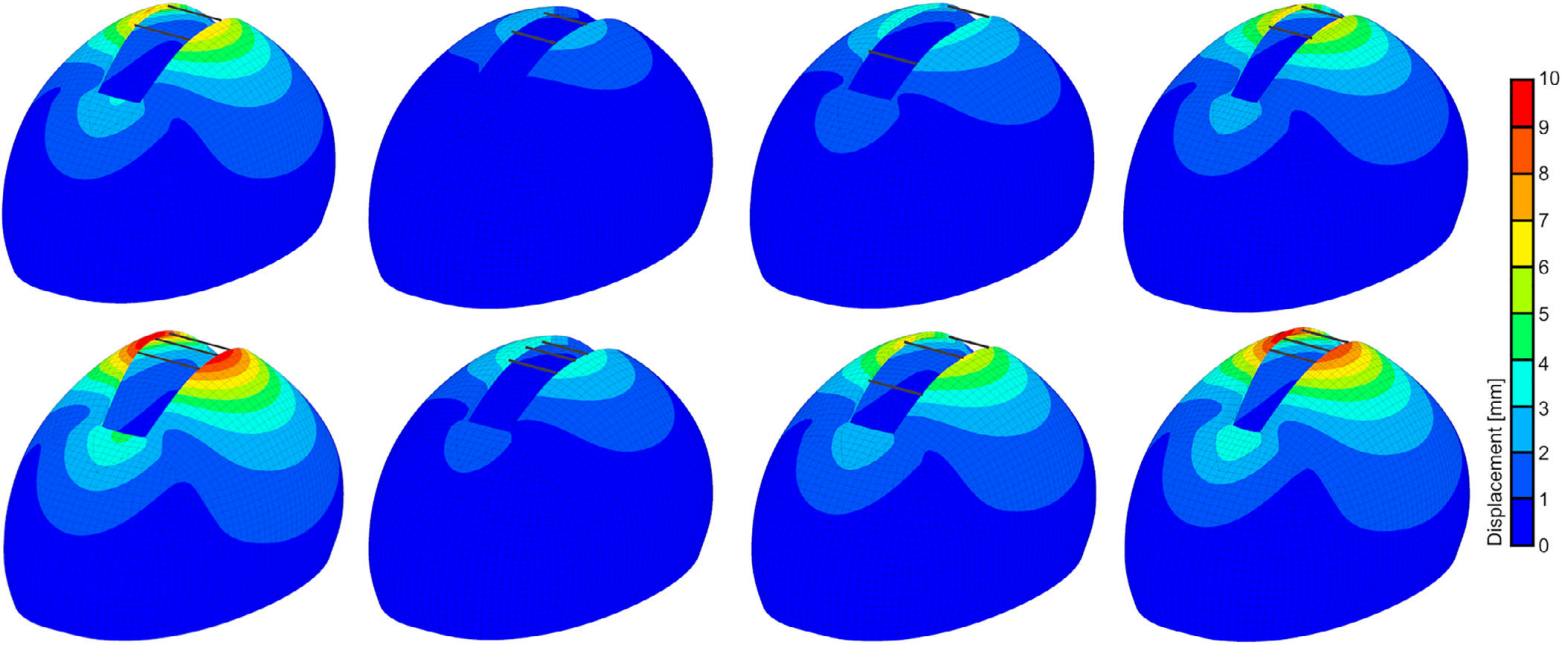
The distribution of the displacements after spring‐assisted cranioplasty in selected skull models.

The absolute difference between the changes in the cranial index due to changes in the spring position and osteotomy size decreases with increasing distance between the springs and sutures and osteotomy size (Figure 5f, l). The distribution of displacements in selected skull models after spring‐assisted cranioplasty is given in Figure 6. Bone, spring, and surgical parameters in the same models are given in Table 4.

**TABLE 4:** Bone, spring and surgical parameters in the selected finite element models simulating spring‐assisted cranioplasty. SAC, *E*, *t*, SP, OS, and *k_s_*, represent spring‐assisted cranioplasty, calvarial bone elastic modulus and thickness, spring position, osteotomy size, and stiffness of the springs, respectively.

—	*E* [MPa]	*t* [mm]	SP [mm]	OS [mm]	*k_s_* [N/mm]
SAC with 2‐spring	300	2	34	20	0.39
	421	3	34	20	0.39
	421	2	19	20	0.39
	421	2	34	12	0.39
SAC with 3‐spring	300	2	34	20	0.39
	421	3	34	20	0.39
	421	2	19	20	0.39
	421	2	34	12	0.39

There were relatively low displacements in the skull models with thicker calvarial bones. Moreover, positioning the anterior and posterior springs relatively close to the sutures again resulted in relatively low displacement in the skull model. The use of three springs to correct the sagittal synostosis caused relatively high displacements for the same parameter values for all the configurations.

## 4 Discussion

Craniosynostosis arises from complex aetiologies and is a growth disorder that remains poorly understood [40]. Recent studies indicate that skull growth is influenced by geometric variations and mechanical factors [41]. Understanding the effects of different factors may help improve surgical results in spring‐assisted cranioplasty. Therefore, in this study, the isolated effects of elastic modulus and thickness of the calvarial bones, spring stiffness and position and osteotomy size on the post‐operative cranial index were evaluated. This involved parameterising the relationships between the elastic modulus and thickness of the calvarial bones, spring position, and osteotomy size, and the post‐operative cranial index through curve fitting. Power functions were used to describe the relations between the post‐operative cranial index and the elastic modulus and thickness of the calvarial bones and spring position, whereas linear functions described the relation between the post‐operative cranial index and the osteotomy size (Figure 2). The relations described by power functions show that the post‐operative cranial index change rate for the skull and surgical parameters reaches a limit value. This also indicates that the change in the post‐operative cranial index may become negligible for the skull or surgical parameters after the limit values. On the other hand, the rate of change in the post‐operative cranial index concerning osteotomy size remains constant and very small. This indicates that varying the osteotomy size will result in a certain increase in the post‐operative cranial index; however, it may be limited. A recent study suggests that head shape may be more sensitive to spring position and osteotomy size in comparison to other surgical parameters [42]. Findings in another study suggest that the mechanical and geometric properties of the skull play a more significant role in the surgical outcome of spring‐assisted correction of sagittal synostosis in comparison to spring and surgical parameters [25]. Jacob and Bozkurt [25] present the overall effects of the skull, spring, and surgical parameters on the surgical outcome using partial regression analyses in statistical shape models. This study evaluates the isolated effects of the skull, spring, and surgical parameters using a parametric skull model and aims to understand how the varying values for skull, spring and surgical parameters affect the surgical outcome. The mean values of the sensitivity indices (Table 3) in this study confirm the findings of [25].

The results of this study also showed that a relatively low elastic modulus in the calvarial bones has a remarkably high influence on the post‐operative cranial index in sagittal synostosis after spring‐assisted cranioplasty. However, a relatively high elastic modulus in the calvarial bones has little effect on the increase in the post‐operative cranial index after spring‐assisted cranioplasty. The thickness of the calvarial bones has similar effects on the increase in the post‐operative cranial index, although the surgical outcome is less sensitive to bone thickness (Table 3). Better results from the craniosynostosis corrective surgeries, including spring‐assisted cranioplasty, are achieved before six months of age [3], whereas mechanical and geometric properties such as elastic modulus and thickness of calvarial bones change with growth [43]. The findings in this study explain why spring‐assisted cranioplasty is more effective in patients younger than six months old.

Direct comparison of the increase in the cranial index after spring‐assisted cranioplasty (Figure 5) shows that a relatively low elastic modulus in the calvarial bones results in a higher post‐operative cranial index in comparison to the other skull, spring, and surgical parameters. However, increasing the elastic modulus results in a minimal increase in the post‐operative cranial index. Bone thickness has a similar effect on the increase in the cranial index after spring‐assisted cranioplasty in comparison with the spring position. These results also show that variations in skull properties may critically affect the surgical outcome. Therefore, surgical planning may require quantifying and considering the skull properties carefully in spring‐assisted cranioplasty.

Positioning the anterior and posterior springs further from the sutures resulted in a relatively high increase in the post‐operative cranial index and in displacements in the skull model after spring‐assisted cranioplasty. This may be because the spring forces affect the mid‐parietal region where the biparietal diameter is measured. It has already been shown that relatively short anterior‐posterior distances between the springs play a crucial role in the head shape [44]. Techniques such as finite element simulations may provide detailed information not only about the cranial index but also the overall head shape depending on the spring positions.

Isolated effects of the spring stiffness on the post‐operative cranial index after spring‐assisted cranioplasty were not analysed using a parametrised model. Because springs have only three different characteristics, which are defined by the wire diameter [36], have been used in the simulations. Using springs with relatively high stiffness resulted in a higher increase in the post‐operative cranial index. Also, using three springs resulted in a relatively high increase in the post‐operative cranial index.

Finite element simulations in this study were performed by varying one parameter and controlling the other parameters using the defined ranges and values presented in Table 1. Therefore, the results in Figure 6 present the effect of varying parameters in each model. Although similar simulations were performed in [23, 25], the results in [23, 25] show randomly combined parameters within a range. Finite element simulations can be used to test the range and decide about the optimal set of parameters on a patient‐specific geometric model reconstructed from computed tomography images. Surgical parameters, such as spring types and sizes, size of the osteotomy, and spring positions can be tested using finite element simulations. The sensitivity analysis results show that the skull's mechanical properties profoundly affect the surgical outcome. A predictive model will require an accurate estimation of the mechanical properties of a patient's skull. Such a task requires further research to find correlations between a patients' age and skull properties in patients with sagittal synostosis. Ajami et al. [24] present an initial investigation of this. Similar research may help develop methods to estimate skull properties in patients. Therefore, there is a need for further research to estimate the mechanical properties of patient skulls with craniosynostosis. Once the patient model is constructed, surgical parameters can be tested as reported in [20, 38, 45].

The finite element simulations were performed as described in the patient‐specific models by implementing the mechanical properties of the bones and sutures, spring forces, and boundary conditions into the model. Patient‐specific simulations replicate the existing procedure, which allows the use of osteotomy sizes and spring forces as in the patient. Because clinical data and pre‐ and post‐op CT images are available as given in [20, 38]. However, this study aims to evaluate the effects of the skull and surgical parameters on the surgical outcome. Therefore, the parameter set was defined within a range. The displacement map in the finite element simulations shows the effect of spring forces on the overall skull geometry. Finite element simulations also provided data for the sensitivity analysis. Finite element simulations allow testing the surgical parameters according to the needs of the patient and the goals of the surgery. Therefore, understanding the effects of the patient's skull parameters and surgical parameters on the surgical outcome is crucial, which is the aim of this study.

In this study, a parametric skull model for sagittal synostosis, as described in [23], was used in the simulations. The sagittal synostosis skull geometry was built considering the average dimensions for the skull length and breadth obtained from statistical shape modelling for 4–6‐month‐old patients [27]. Borghi et al. [46] investigate the relationship between the perception of craniofacial deformity and geometric head features and perform an analysis of 3D head shapes using statistical shape modelling. They describe the section and guidelines for the skull as used in the skull geometry used in this study. Also, data reported in [15] show that there is high variation in the dimensions of the skulls with sagittal synostosis. The dimensions of the sagittal synostosis skull model remain within the reported range, resulting in an anatomical cranial index for a five‐month‐old infant with sagittal synostosis. Therefore, the overall shape and dimensions of the skull geometry used in this study represent sagittal synostosis accurately.

This study had the following limitations. The thickness of the calvarial bones was uniform. However, there are regional variations in skull bone thickness in children [32]. The growth of the skull was not included in the simulations. Nonetheless, data in the literature show that skull growth may not influence the post‐operative cranial index [8, 47]. The viscoelastic properties of the calvarium were not simulated. Viscoelasticity shows the effect of the springs on the skull over time [20]. Simulation results and performed analyses show the immediate effects of spring‐assisted cranioplasty on surgical outcomes in sagittal synostosis. Nonetheless, the isolated effects of the springs on the surgical outcomes after spring‐assisted cranioplasty were not analysed using parametrised models. The effects of the skull, spring and surgical parameters on the surgical outcomes after spring‐assisted cranioplasty were evaluated only for one skull model. Therefore, the results are valid for the skull model in this study, however, similar relations between the increase in the post‐operative cranial index after spring‐assisted cranioplasty and skull, spring, and surgical parameters are also expected for different skull geometries affected by sagittal synostosis.

## 5 Conclusions

Simulation results and sensitivity indices showed that relatively low elastic modulus and thickness in the calvarial bones has remarkable effects on surgical outcomes in sagittal synostosis after spring‐assisted cranioplasty. However, the post‐operative cranial index after spring‐assisted cranioplasty increases a little for higher elastic modulus and thickness in the calvarial bones. Therefore, evaluating the mechanical and geometric properties of the skull during surgical planning may ensure successful surgical outcomes in sagittal synostosis treated with spring‐assisted cranioplasty. Further research will help to develop methods for this, and the future, it may be possible to use skull properties accurately in predictive models to plan surgeries in craniosynostosis patients.

## Author Contributions

**Jenson Jacob**: conceptualization, formal analysis, investigation, methodology, software, validation, writing – original draft. **Selim Bozkurt**: conceptualization, data curation, formal analysis, investigation, methodology, project administration, resources, software, supervision, validation, writing – original draft, writing – review and editing.

## Funding

The authors have nothing to report.

## Conflicts of Interest

The authors declare no conflicts of interest.

## Data Availability

Data is available upon reasonable request from the corresponding author.
